# A randomised phase II trial of docetaxel *vs* docetaxel and irinotecan in patients with stage IIIb–IV non-small-cell lung cancer who failed first-line treatment

**DOI:** 10.1038/sj.bjc.6602268

**Published:** 2004-12-14

**Authors:** F M Wachters, H J M Groen, B Biesma, F M N H Schramel, P E Postmus, J A Stigt, E F Smit

**Affiliations:** 1 Department of Pulmonary Diseases, University Hospital Groningen, PO Box 30.001, 9700 RB Groningen, the Netherlands; 2 Department of Pulmonary Diseases, Jeroen Bosch Hospital, PO Box 90.153, 5200 ME s Hertogenbosch, the Netherlands; 3 Department of Pulmonary Diseases, St Antonius Hospital, PO Box 2500, 3430 EM Nieuwegein, the Netherlands; 4 Department of Pulmonary Diseases, Vrije Universiteit Medical Center, PO Box 7057, 1007 MB Amsterdam, the Netherlands; 5 Department of Pulmonary Diseases, Isala Clinics, PO Box 10.400, 8000 GK Zwolle, the Netherlands; 6 Department of Pulmonary Diseases, Martini Hospital, PO Box 30.033, 9700 RM Groningen, the Netherlands

**Keywords:** carcinoma, non-small-cell lung, clinical trial, phase II, docetaxel, irinotecan, second-line chemotherapy

## Abstract

Response rate and toxicity of second-line therapy with docetaxel (75 mg m^−2^) or docetaxel, irinotecan, and lenogastrim (60 mg m^−2^, 200 mg m^−2^, and 150 *μ*g m^−2^ day^−1^, respectively) were compared in 108 patients with stage IIIb–IV non-small-cell lung cancer. Addition of irinotecan to docetaxel does not improve response rate, and increases gastrointestinal toxicity.

Treatment with platinum-based chemotherapy improves survival and quality of life in patients with advanced non-small-cell lung cancer (NSCLC) (Souquet *et al*, 1993; Non-small Cell Lung Cancer Collaborative Group, 1995; Cullen *et al*, 1999; Anderson *et al*, 2000; Ranson *et al*, 2000; Schiller *et al*, 2002). Since two trials demonstrated clinically beneficial effects of docetaxel in second-line setting (Fossella *et al*, 2000; Shepherd *et al*, 2000), docetaxel 75 mg m^−2^ is currently considered the standard regimen to which other experimental schedules should be compared.

Irinotecan, a semisynthetic water-soluble analogue of camptothecin, has shown activity in NSCLC patients as single agent and in combination with docetaxel (Fukuoka *et al*, 1992; Adjei *et al*, 2000; Masuda *et al*, 2000; Satouchi *et al*, 2001; Negoro *et al*, 2003). Moreover, Irinotecan demonstrated activity as single agent in pretreated patients (Sanchez *et al*, 2003). In our trial the efficacy of the combination of docetaxel and irinotecan compared to single-agent docetaxel as second-line treatment in NSCLC was investigated. Primary end point of this randomised phase II study was tumour response rate. Secondary end points were toxicity, progression-free survival and overall survival. The treatment regimen was based on a phase II study, which demonstrated activity of docetaxel and irinotecan in patients with recurrent ovarian cancer (Mäenpää *et al*, 1999). In this study, neutropenia was the main toxicity; therefore, we added a granulocyte colony-stimulating factor to our treatment regimen.

## PATIENTS AND METHODS

### Patient selection

Inclusion criteria for enrolment in the trial were age ⩾18 years, stage IIIb–IV NSCLC, failure or relapse after first-line chemotherapy, at least one measurable or evaluable tumour lesion, performance status ⩽2 according to the Eastern Cooperative Oncology Group scale, life expectancy of ⩾3 months, adequate bone marrow reserve (neutrophils ⩾1.5 × 10^9^ l^−1^, platelets ⩾100 × 10^9^ l^−1^, haemoglobin ⩾6.2 mmol l^−1^), renal function (serum creatinine ⩽1.25 times the upper normal limit), and liver function (serum bilirubin⩽the upper limit of the institutional reference value, serum alanine aminotransferase (ALAT) and serum aspartate aminotransferase (ASAT) ⩽2.5 times the upper normal limit, alkaline phosphatase ⩽5 times the upper normal limit). Prior radiotherapy was allowed as long as the irradiated area did not contain the sole measurable or evaluable lesion. Exclusion criteria were active infection, second primary malignancies (except carcinoma *in situ* of the cervix, adequately treated basal cell carcinoma of the skin, and other cancer curatively treated without recurrence for at least 5 years), symptomatic brain metastases, inflammatory bowel diseases, symptomatic peripheral neuropathy ⩾grade 2 according to the Common Toxicity Criteria (CTC) of the National Cancer Institute (version 2.0), serious cardiac diseases, contraindications for use of corticosteroids, pregnancy, breast-feeding, or reproductive potential without implementing adequate contraceptive measures. Local medical ethics committees of all hospitals approved the protocol. All patients gave informed consent.

### Treatment

Patients were randomised by block randomisation to receive either docetaxel 75 mg m^−2^ on day 1 (D arm) or docetaxel 60 mg m^−2^ and irinotecan 200 mg m^−2^ both on day 1 followed by lenogastrim 150 *μ*g m^−2^ day^−1^ on days 2–12 (DI arm). Docetaxel (in 250 ml 0.9% NaCl) was administered as a 1-h intravenous infusion in both treatment arms. Irinotecan (3 mg ml^−1^, diluted with 0.9% NaCl) was administered after docetaxel as a 90-min infusion. Lenogastrim ampoules contained 263 *μ*g recombinant human granulocyte colony-stimulating factor dissolved in 1 ml solvent for subcutaneous injection. Treatment was repeated every 3 weeks for a maximum of five cycles and halted in case of tumour progression, intolerable toxicity or patient's wish. To prevent hypersensitivity reactions caused by docetaxel dexamethason 8 mg was given twice a day during 3 subsequent days starting the day before infusion. Antiemetics consisted of ondansetron 8 mg twice a day on days 1–3. In case of diarrhoea, patients were treated with loperamide (starting dose 4 mg, followed by 2 mg every 2 h as long as diarrhoea continued, maximum dose 16 mg per day). When the diarrhoea persisted for more than 48 h or occurred in combination with neutropenia, fever or dehydration, patients were hospitalised and treated with antibiotics.

### Dose adjustments

Drug administration was postponed (maximally 2 weeks) if there was no haematologic recovery on day 22 (leukocytes <3.0 × 10^9^ l^−1^ and/or platelets <100 × 10^9^ l^−1^). In case of nadir values of neutrophils <0.5 × 10^9^ l^−1^ or platelets <50 × 10^9^ l^−1^ exceeding 7 days, febrile neutropenia, or thrombocytopenia associated with bleeding, the dose of docetaxel for subsequent cycles was reduced to 55 mg m^−2^ in the D arm and to 45 mg m^−2^ in the DI arm. The dose of irinotecan was reduced to 150 mg m^−2^ in these cases. In the event of grade 3–4 nonhaematologic toxicity (except nausea and vomiting) or grade 2 neuropathy, the doses of docetaxel and irinotecan were reduced with 25% for subsequent cycles. Treatment was stopped if the same severity of toxicity occurred at the reduced dose level treatment or in case of grade 3–4 neuropathy. In case of grade 3–4 diarrhoea lasting more than 2 weeks despite appropriate therapy, no further irinotecan was administered.

### Treatment evaluation

Complete blood cell counts were performed weekly during treatment. On day 1 of each cycle, patient evaluation also included liver and renal functions, performance status, chest X-ray, and toxicity scoring according to CTC. All patients were evaluable for toxicity. Tumour response was evaluated according to World Health Organisation criteria (1979).

After discontinuation of treatment, physical examination, laboratory tests, chest X-ray, and additional imaging tests on clinical indication to assess tumour progression were performed every 6 weeks.

### Statistical analysis

The ‘pick the winner’ format based on the randomised phase II clinical trials approach as proposed by Simon *et al* (1985) was used. In this approach, an accrual of 53 patients in each arm gives a 90% chance of selecting the better treatment schedule if the difference in response rate is at least 10% and the smaller response rate is assumed to be approximately 15%. The arm with the highest response rate is declared the ‘winner’ providing that the response rate is at least 15%. A statistically significant difference in response rate is not required in this trial design. This trial design is not suitable to test hypotheses of equality of effects. Moreover, with this approach, a treatment can still be selected even if the difference in response rate is less than 10%, but then the probability of correct selection will not be as great as 90%.

In order to terminate an ineffective schedule early in the study a three-stage stopping rule was used, as proposed earlier by Hoskins *et al* (1998). The conclusion from this study would be based on the ranking of the response rate. No formal statistical comparison between the two arms was planned for the primary end point.

Patient characteristics, treatment parameters, and toxicity in both arms were compared using Student's *t*-test, Mann–Whitney test, *χ*^2^ test, or Fisher's exact test. The time from the date of randomisation to the date of first documented progression was defined as progression-free survival. Overall survival was defined as the interval between the date of randomisation and the date of death. Survival data were compared by Kaplan–Meier curves using the log-rank test. A *P*<0.05 was considered statistically significant.

## RESULTS

### Patient characteristics

Between October 2000 and January 2003, 108 patients from five hospitals in the Netherlands were randomised to D (*n*=56) or DI (*n*=52). Patient characteristics were not significantly different between both treatment arms (Table 1).

### Toxicity

Haematologic toxicity is shown in Table 2. In the D arm, grade 3 or 4 leukopenia and granulocytopenia occurred more frequently as compared to the DI arm. However, in both arms, an equal number of patients was hospitalised for febrile neutropenia. Significantly more patients in the DI arm had thrombocytopenia. Anaemia occurred in both arms at equal frequency. At the time of study, erythropoietin was not routinely administered.

The worst nonhaematologic toxicity per patient is listed in Table 3. Diarrhoea was more frequently observed in the DI arm (*P*<0.01). In this arm, six patients were hospitalised for serious diarrhoea compared to one patient in the D arm (*P*=0.05). Nail changes and arthralgia were only observed in the D arm, where the higher docetaxel dose was administered. Additionally, significantly more patients in the D arm had myalgia (*P*<0.05).

### Treatment

A total of 206 and 162 cycles were administered in the D and DI arm, respectively. The median (range) number of cycles per patient was 4 (1–5) in the D arm, and 3 (1–5) in the DI arm. The maximum of five cycles was completed in 25 (45%) patients in the D arm and in 17 (33%) patients in the DI arm (*P*>0.05). The reasons for treatment discontinuation for both arms were not different. Main reasons for treatment discontinuation were progressive disease and toxicity. Seven patients died due to disease progression while on protocol therapy. Three patients died due to toxicity of treatment, due to paralytic ileus after one cycle (*n*=1; D arm), bowel perforation after two cycles (*n*=1; DI arm), and myocardial infarction after one cycle (*n*=1; DI arm). Doses of docetaxel (D arm), docetaxel (DI arm), and irinotecan had to be reduced in 4, 2, and 7% of the cycles, respectively. In both arms, drug administration was postponed up to 2 weeks in 2% of the cycles.

### Tumour response

At the end of the first stage, one response was observed in the D arm *vs* two in the DI arm. A total of five responses was observed in both arms at the end of the second stage. Subsequently, enrolment continued to a total of 108 patients. Final analysis revealed an overall response rate of 16% (95% CI, 6–26) for the D arm and 10% (95% CI, 2–18) for the DI arm (Table 4). According to the statistical design of this trial, the D arm was declared the ‘winner’. Nine patients were not evaluable for tumour response due to early death (*n*=1; D arm, *n*=2; DI arm), discontinuation of treatment at patients request (*n*=1; D arm), and discontinuation for toxicity (*n*=5; DI arm). These patients were considered nonresponders.

### Progression-free survival and overall survival

**Figure 1:**
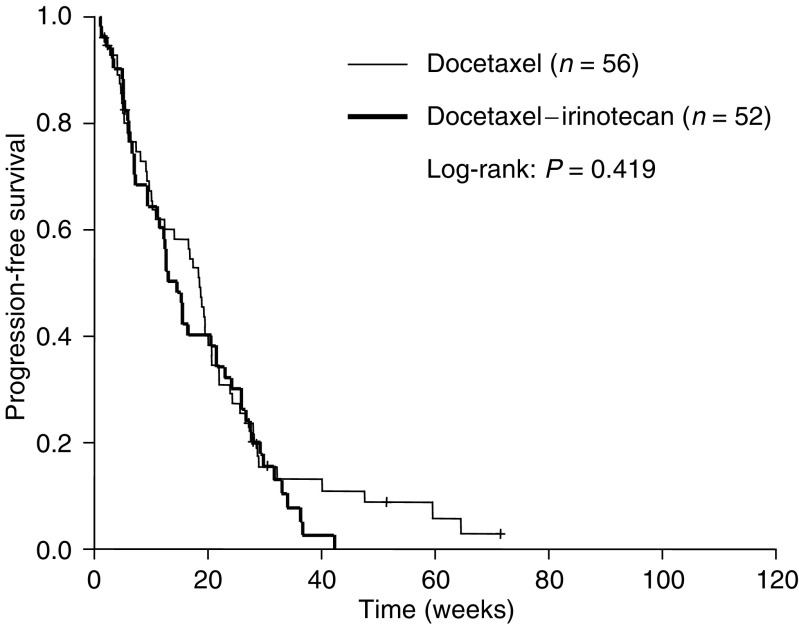
Progression-free survival after second-line chemotherapy in advanced NSCLC.

**Figure 2:**
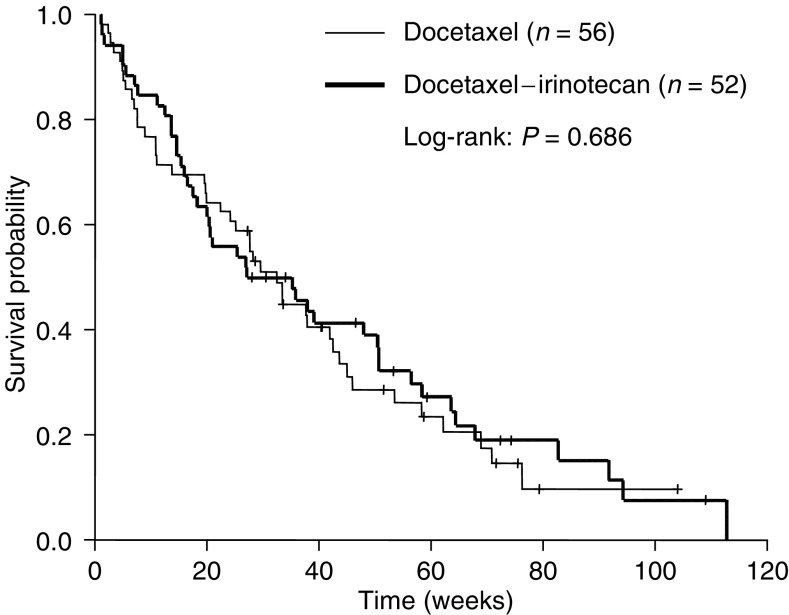
Overall survival after second-line chemotherapy in advanced NSCLC.

In August 2003, 22 patients were still alive. The median progression-free survival was not significantly different between both treatment arms; 18 (95% CI, 16–21) *vs* 15 (95% CI, 12–18) weeks for the D and DI arm, respectively (*P*=0.42) (Figure 1). The median overall survival was 32 (95% CI, 25–40) weeks in the D arm, *vs* 27 (95% CI, 8–46) weeks in the DI arm, which was not different between both arms (*P*=0.69). The 1-year survival rate (±s.e.) was 26% (±6%) *vs* 30% (±7%) in the D and DI arm, respectively (*P*=0.49) (Figure 2).

## DISCUSSION

According to the statistical design of this trial, the docetaxel arm, with a response rate of 16%, ranked superior compared to the docetaxel–irinotecan arm, with a response rate of 10%. Using this design, smaller number of patients are required compared to the usual randomised trial design in which sample size calculations are based on statistical significance. The conclusion of this trial – that addition of irinotecan to docetaxel as second-line chemotherapy in NSCLC does not improve response rate – is solely based on the ranking of response rate. Although our trial was not powered to detect differences in survival, the efficacy of docetaxel as single agent and docetaxel combined with irinotecan seemed not different in terms of progression-free survival and overall survival.

Both arms showed a different toxicity profile. Leukopenia, nail changes, myalgia, and arthralgia occurred more frequently in the D arm, while thrombocytopenia and diarrhoea were more common in the DI arm. Toxicities especially occurring in the D arm might probably be related to the higher dose level of docetaxel used in this arm. Nevertheless, toxicity in the docetaxel arm was acceptable. As in other trials, diarrhoea frequently occurred after treatment with docetaxel and irinotecan (Adjei *et al*, 2000; Masuda *et al*, 2000; Satouchi *et al*, 2001). In the majority of patients, in this trial, diarrhoea resolved after treatment with loperamide. An option for prevention of delayed-type diarrhoea (occurring >24 h after irinotecan administration) is administration of the poorly absorbed aminoglycoside antibiotic neomycin, which decreases the intestinal SN-38 (active metabolite of irinotecan) concentration by inhibition of *β*-glucuronidase activity from intestinal microflora (Kehrer *et al*, 2001).

The response rate for docetaxel monotherapy found in this trial is comparable to results of other phase II trials, which found a response rate between 16–22% (Fossella *et al*, 1995; Gandara *et al*, 2000; Robinet *et al*, 2000). In two phase III trials a lower response rate (7–11%) was reported (Fossella *et al*, 2000; Shepherd *et al*, 2000). Median survival for docetaxel monotherapy in our study was 7.5 months. The two mentioned phase III trials reported a median survival of 7.5 and 5.7 months, respectively (Fossella *et al*, 2000; Shepherd *et al*, 2000).

Two other single agents were recently investigated in a second-line setting in NSCLC patients. Gefitinib, an orally active EGFR tyrosine kinase inhibitor, was studied in a randomised phase II trial (Fukuoka *et al*, 2003). Antitumour activity, with a response rate between 18 and 19%, as well as symptom relief and improvement in quality of life was reported. Median survival was between 7.6 and 8.0 months. Pemetrexed, a novel multitargeted antifolate, was compared to single-agent docetaxel in a recently published phase III trial (Hanna *et al*, 2004). No significant differences in response rate and survival were found between patients in both arms. Response rate and survival were in accordance with other data on single-agent docetaxel as second-line treatment (Fossella *et al*, 2000; Shepherd *et al*, 2000). However, in the pemetrexed arm less toxicity, especially febrile neutropenia, was observed. Therefore, pemetrexed can probably be used as alternative reference regimen in the second-line treatment of advanced NSCLC.

Whether combinations are superior to single agents in second-line setting is presently unclear. Although this trial demonstrated no clear benefit using docetaxel with irinotecan, other regimens using the combination of these drugs with filgrastrim support or with celecoxib are currently under investigation (Argiris, 2003; Frasci *et al*, 2004).

In conclusion, addition of irinotecan to docetaxel as second-line chemotherapy does not improve response rate, and increases gastrointestinal toxicity in patients with stage IIIb or IV NSCLC.

## Figures and Tables

**Table 1:** Patient characteristics

	**Docetaxel**		**Docetaxel–Irinotecan**		
	**No.**	**%**	**No.**	**%**	***P***
Patients entered	56		52		NS
*Age (years)*					NS
Median	59		58		
Range	36–78		42–76		
			
*Sex*					NS
Male	44	79	32	62	
Female	12	21	20	38	
					
*Stage*					NS
IIIb	14	25	11	21	
IV	42	75	41	79	
					
*Performance status*					NS
0	10	18	13	25	
1	39	70	37	71	
2	7	13	2	4	
					
*Histology*					NS
Squamous cell carinoma	17	30	10	19	
Adenocarcinoma	25	45	22	42	
Large cell carcinoma	11	20	19	37	
Other	3	5	1	2	
					
*Previous radiotherapy*					
To primary tumour	16	29	14	27	NS
To metastases	10	18	4	8	NS
					
*First-line chemotherapy*					NS
Platinum-based	40	71	39	75	
Nonplatinum-based	16	29	13	25	
					
*RR to first-line chemotherapy*					NS
%	54		48		
95% CI	41–67		34–62		
			
*Treatment interval (weeks)* a					NS
Median	35		29		
Range	2–167		1–147		

NS=not significant, RR=response rate, CI=confidence interval.

a Interval between completion of previous chemotherapy and the date of randomisation.

**Table 2:** Worst haematologic CTC toxicity grade per patient for all cycles

	**Docetaxel**		**Docetaxel–Irinotecan**		
**Toxicity**	**No.**	**%**	**No.**	**%**	***P***
*Anaemia*					NS
1	39	70	28	54	
2	11	20	14	30	
3/4	0	0	5	10	
					
*Leukopenia*					<0.05
1	6	11	6	12	
2	16	29	7	13	
3/4	16	29	7	13	
					
*Granulocytopenia* a					<0.05
1	1	4	2	7	
2	7	25	1	4	
3/4	12	43	6	22	
					
*Thrombocytopenia*					<0.05
1	5	9	13	25	
2	1	2	2	4	
3/4	0	0	2	4	
					
Febrile neutropenia	3	5	3	6	NS

a Neutrophil granulocyte counts were only available in 55 patients. NS=Not significant.

**Table 3:** Worst nonhaematologic CTC toxicity grade per patient for all cycles

	**Docetaxel**		**Docetaxel–Irinotecan**		
**Toxicity**	**No.**	**%**	**No.**	**%**	***P***
*ASAT*					NS
1	6	11	4	8	
2	1	2	0	0	
3	0	0	1	2	
					
*ALAT*					NS
1	20	36	18	35	
2	4	7	3	6	
					
*Creatinine*					NS
1	21	38	8	15	
2	1	2	3	6	
3	0	0	1	2	
					
*Mucositis*					NS
1	8	14	5	10	
2	1	2	0	0	
					
*Nausea*					NS
1	15	27	14	27	
2	5	9	12	23	
					
*Vomiting*					NS
1	11	20	9	17	
2	4	7	4	8	
					
*Diarrhoea*					<0.01
1	11	20	21	40	
2	4	7	11	21	
3	1	2	7	14	
					
*Fever*					NS
1	5	9	2	4	
2	2	4	0	0	
					
*Infections*					NS
1	3	5	2	4	
2	0	0	2	4	
3	1	2	0	0	
					
*Alopecia*					<0.05
1	15	27	6	12	
2	16	29	25	48	
					
*Skin reactions*					NS
1	2	4	0	0	
2	3	5	0	0	
					
*Sensory neuropathy*					NS
1	13	23	10	19	
2	4	7	1	2	
					
*Motoric neuropathy*					NS
1	2	4	0	0	
2	0	0	0	0	
3	0	0	1	2	
					
*Anorexia*					NS
1	16	29	16	31	
2	4	7	4	8	
					
*Fatigue*					NS
1	23	41	13	25	
2	12	21	18	35	
					
*Nail changes*					0.052
1	5	9	0	0	
2	1	2	0	0	
					
*Myalgia*					<0.05
1	12	21	3	6	
2	2	4	0	0	
					
*Arthralgia*					<0.05
1	5	9	0	0	

NS=Not significant.

**Table 4:** Tumour response after second-line chemotherapy in advanced NSCLC

	**Docetaxel**		**Docetaxel–Irinotecan**	
**Response**	**No.**	**%**	**No.**	**%**
Complete response	1	2	0	0
Partial response	8	14	5	10
Stable disease	25	45	22	42
Progressive disease	20	36	18	35
Not assessable	2	4	7	14
				
Overall response (95% CI)		16 (6–26)		10 (2–18)
